# Use of Etomidate for Rapid Sequence Intubation (RSI) in Pediatric Trauma Patients: An Exploratory National Survey †

**DOI:** 10.3390/pharmacy3040197

**Published:** 2015-10-19

**Authors:** Jeffrey J. Cies, Matthew L. Moront, Wayne S. Moore, Renata Ostrowicki, Kelsey B. Gannon, Shonola S. Da-Silva, Arun Chopra, Jason Parker

**Affiliations:** 1 St. Christopher’s Hospital for Children, Philadelphia, PA, 19134, USA; E-Mails: matthew.morontmd@tenethealth.com (M.L.M.); Renata.ostrowicki@tenethealth.com (R.O.); Kelsey.gannon@tenethealth.com (K.B.G.); Shonola.da-silvamd@tenethealth.com (S.S.D.-S.); Jason.parker@tenethealth.com (J.P.); 2 Drexel University College of Medicine, Philadelphia, PA, 19129, USA; 3 Alfred I duPont Hospital for Children, Wilmington, DE, 19803, USA; E-Mail: wayne.mooreII@nemours.org; 4 NYU Langone Medical Center, New York, NY, 10016, USA; E-Mail: arun.chopra@nyumc.org; 5 NYU School of Medicine, New York, NY, 10016, USA

**Keywords:** etomidate, adrenal suppression, pediatric, trauma RSI

## Abstract

*Objective*, To survey the pediatric trauma programs to ascertain if and how etomidate is being used for rapid sequence intubation (RSI) in pediatric trauma patients. *Design*, A 25 question survey was created using REDCaps. A link to the survey was emailed to each of the pediatric and adult trauma programs that care for pediatric patients. *Setting*, Pediatric trauma programs and adult trauma programs caring for pediatric patients. *Intervention*, None. *Measurements and Main Results*, A total of 16% of programs responded (40/247). The majority of the centers that responded are urban, academic, teaching Level 1 pediatric trauma centers that provide care for > 200 pediatric trauma patients annually.  The trauma program directors were the most likely to respond to the survey (18/40).  33/38 respondents state they use etomidate in their RSI protocol but it is not used in all pediatric trauma patients.  26/38 respondents believe that etomidate is associated with adrenal suppression and 24/37 believe it exacerbates adrenal suppression in pediatric trauma patients yet 28 of 37 respondents do not believe it is clinically relevant. *Conclusions*, Based on the results of the survey, the use of etomidate in pediatric trauma patients is common among urban, academic, teaching, level 1 pediatric trauma centers.  A prospective evaluation of etomidate use for RSI in pediatric trauma patients to evaluate is potential effects on adrenal suppression and hemodynamics is warranted.

## 1. Introduction

Pediatric trauma patients are often intubated using rapid sequence intubation (RSI) with an anesthetic and paralytic agent. Induction agents include narcotics, benzodiazepines, non-benzodiazepine hypnotics, and barbiturates. Etomidate is an imidazole-derived ultra-short acting non-barbiturate hypnotic [1]. Potential advantages often cited when using etomidate are rapid onset, lack of significant cardiovascular effects, limited respiratory depression, lack of histamine release, and a short duration of action [1]. However, etomidate has been shown to inhibit the 11-beta-hydroxylase enzyme which prevents conversion of deoxycortisol to cortisol in the adrenal gland [2]. This can lead to decreased concentrations of cortisol for up to 48–72 h after administration, sometimes causing profound suppression of cortisol secretion [2].

Continuous intravenous administration of etomidate has been associated with adrenocortical dysfunction (ACD) and increased patient mortality in adults [3,4,5,6]. Several small randomized clinical studies have evaluated the use of single dose intravenous administration of etomidate as an induction agent for endotracheal intubation in elective surgery patients [7,8,9,10,11,12,13,14,15,16,17,18,19,20,21,22], emergency room patients [23,24], and the critically ill [25,26,27]. These studies have demonstrated transient (e.g., <48 h) suppression of adrenocortical function after single dose administration. Several adult studies have also suggested that etomidate leads to adrenal insufficiency in trauma patients [28,29,30] and increases mortality in adult patients with sepsis and septic shock [31,32]. Furthermore, the use of etomidate has been associated with an increased mortality in children with meningococcal sepsis [33,34]. The use of etomidate for RSI in the pediatric trauma population is not well described and could be affecting morbidity and mortality in this population. As such, this justifies further research and investigation regarding the role of etomindate for RSI in this population. Therefore, the primary objective of this exploratory survey was to describe the use of etomidate for RSI in pediatric trauma patients as a means to determine the current role of etomidate in this population for potential future research opportunities.

## 2. Materials and Methods 1

A 25 question survey (Appendix 1) was created using REDCap [35]. Study data were collected and managed using REDCap electronic data capture tools hosted at Drexel University College of Medicine. REDCap (Research Electronic Data Capture) is a secure, web-based application designed to support data capture for research studies, providing: (1) an intuitive interface for validated data entry; (2) audit trails for tracking data manipulation and export procedures; (3) automated export procedures for seamless data downloads to common statistical packages; and (4) procedures for importing data from external sources [35]. The names of all trauma programs in the United States that care for pediatric trauma patients was obtained from the American Trauma Society (ATS) as was the contact information for the given trauma program. A link to the survey was emailed to the contact person listed on file at the ATS for each of the Pediatric trauma programs and the Adult trauma programs that care for pediatric patients. The survey was conducted anonymously with the exception that the respondent was asked the name of the hospital or program for which they were providing answers so that duplicate responses were not provided. Two additional emails were sent at 3 months and 5.5 months with reminders to complete the online survey. After 6 months, the survey link was de-activated and the information was downloaded for analysis. The survey was approved for use by the Drexel University College of Medicine Institutional Review Board.

## 3. Results

Correspondence regarding the survey was sent to a total of 247 programs that provide care for pediatric trauma patients in the United States. A total of 40 responses were received for a response rate of 16.2%. The majority of the centers, (20/40, 50%), that responded were Level 1 academic, teaching, pediatric trauma centers that describe themselves as located in an urban (20/40, 50%) setting that provide care for >200 (32/40, 80%) pediatric trauma patients annually (Table 1). The trauma program director was the most likely individual to respond to the survey, 18 of 40 respondents (45%).

Fifteen (15/40, 37.5%) respondents indicated that between 25 and 49 pediatric trauma patients are intubated annually followed by 13 (13/40, 32.5%) respondents indicated they intubate >50 pediatric trauma patients annually. Twenty-three (23/40, 57.5%) respondents state they provide care for pediatric trauma patients in a separate trauma bay within the emergency department (ED) and the airway is primarily managed by emergency department (ED) personnel (25/39, 64%, 1 non-response) followed by anesthesia (9/39, 23%, 1 non-response) (Table 2). Thirty-three of 38 (86.8%, two non-responses) respondents state etomidate is used in their RSI protocol and 14 of 40 (35%) respondents use etomidate regardless of presenting etiology. Twenty-six of 38 (68.4%, two non-responses) respondents believe that etomidate is associated with adrenal suppression and 24 of 37 (64.8%, three non-responses) believe it exacerbates adrenal suppression in pediatric trauma patients. However, 28 of 37 (75.7%, 3 non-responses) respondents do not believe the adrenal suppression induced by etomidate is clinically relevant although 24 (24/37, 65%, 3 non-responses) respondents state that etomidate can worsen the adrenal suppression commonly seen in pediatric trauma patients at baseline. No respondents check cortisol levels in their pediatric trauma patients.

**Table 1:** Survey Respondent Demographics.

**Geographic Region**	
Northeast	5 (12.5%)
Southeast	5 (12.5%)
Midwest	17 (42.5%)
West	9 (22.5%)
Southwest	4 (10%)
**Description**	
Academic	28(70%)
Non-Academic	5(12.5%)
Teaching	22 (55%)
Non-Teaching	1(2.5%)
Urban	20 (50%)
Rural	1(2.5%)
Suburban	1(2.5%)
**Certification**	
Level 1 Pediatric	20 (50%)
Level 2 Pediatric	9 (22.5%)
Level 1 Adult with Pediatric Certification	9 (22.5%)
Level 2 Adult with Pediatric Certification	2 (5%)
**Survey Respondent Role**	
Trauma Director	18 (45%)
Trauma Coordinator	10 (25%)
Anesthesiologist	1 (2.5%)
ED Director	6 (15%)
Other	5 (12.5%)
**Annual Traumas**	
50–100	2 (5%)
>100–200	6 (15%)
>200	32 (80%)
**Annual Pediatric Intubations**	
<10	2 (5%)
10 to 24	10 (25%)
25 to 49	15 (37.5%)
> 50	13 (32.5%)
**Area Pediatric Patient Cared For**	
Separate Trauma Bay	23 (57.5%)
General ED	17 (42.5%)

**Table 2:** Survey Responses.

**Who Manages the Airway**	
Critical Care	1 (2.6%)
ED	25 (64.1%)
Anesthesia	9 (23.1%)
Other	4 (10.3%)
**Primary Advantage of Etomidate**	
Rapid onset	8 (25%)
Permits neurologic evaluation sooner	3 (9.4%)
Minimal hemodynamic effect	17 (53.1%)
Drug of choice for TBI	2 (6.3%)
Other	2 (6.3%)
**Primary Disadvantage of Etomidate**	
Nausea/vomiting	1 (6.7%)
Laryngospasm	1 (6.7%)
Adrenal suppression	12 (80%)
Other	1 (6.7%)

## 4. Discussion

The survey response rate of 16.2% was low but not unexpected when surveying medical and program directors [36]. Within the respondents, the use of etomidate for RSI in pediatric trauma patients appears to be common among the respondents in this investigation. The use of etomidate was similar regardless of the geographic location, trauma program accreditation or academic/teaching status of the respondent. Similarly, etomidate use did not appear to be different as to who managed the airway for intubation, the number of total pediatric trauma patients or the number of pediatric trauma patients requiring intubation.

Despite the potential concern(s), etomidate use in RSI appears to be more frequent than originally expected. One of the most concerning adverse effects with etomidate is a reduction in cortisol production resulting from inhibition of 11-β-hydroxylase within the adrenal cortex [2]. ACD was initially reported in a study of critically ill adults by Ledingham and Watt in 1983 where repeated or continuous administration of etomidate increased the risk for adrenal suppression [3]. Studies conducted after a single etomidate dose have shown mixed results. In 1998, Donmez evaluated plasma cortisol levels in children undergoing cardiac surgery [37]. Thirty children (aged 1 to 11 years) were randomized to receive either 0.3 mg/kg of etomidate or 1 mg/kg of ketamine for induction and all patients received 1 mcg/kg of fentanyl. Plasma cortisol levels were measured at the following time points; baseline, after induction, after cross-clamping, at the end of surgery, and 24 h after surgery. Within the etomidate group, the cortisol levels remained significantly lower than baseline at all post-induction measurements. When comparing the etomidate group to the ketamine group, the cortisol levels were significantly lower in the etomidate group when compared to the ketamine group at all time points post-inductions (*p* < 0.05). In a retrospective study, den Brinker found a similar decrease of cortisol levels in children with meningococcal sepsis given a single dose of etomidate for RSI [33]. Sixty children with meningococcal sepsis had their adrenocortical function evaluated. Twenty-three (38.3%) of the children received etomidate. The children given etomidate had significantly lower cortisol levels at 12 and 24 h as well as higher adrenocorticotropin hormone (ACTH) and 11-deoxycortisol levels than children who did not receive etomidate. The authors concluded that a single dose of etomidate may suppress cortisol production for at least 24 h in children. Further, they speculate a correlation that increased the risk of death in children with meningococcal sepsis. While each of these studies demonstrated a reduction in cortisol levels, the clinical significance of this decrease remains controversial.

In 2000, Sokolove reviewed 100 children <10 years of age who received etomidate for RSI [38]. No children received corticosteroid replacement for suspected ACD during their hospitalization, supporting the authors’ contention that a single dose of etomidate would not routinely produce clinically significant ACD. In 2006, Zuckerbraun *et al.* published a review of 77 children that received etomidate for RSI in a pediatric ED [39]. The mean age was 8.2 ± 6.2 years with a mean etomidate dose of 0.31 ± 0.07 mg/kg (range 0.05–0.64 mg/kg). Adjunctive medications included lidocaine, atropine, and rocuronium and were used in 76 of the 77 children. The authors judged the intubating conditions to be “good” in 68 of 69 patients who had an assessment documented. Generally, etomidate produced a 10% mean decline in systolic blood pressure, a decline in blood pressure of 20% or greater was noted in only 12 (17.4%) patients and based on these results, the authors suggested that etomidate may be a useful agent for RSI in children, but requires further study. Further, the Society of Critical Care Medicine recommends that etomidate not be used as a part of RSI for adult or pediatric patients when there is a concern for sepsis [40].

The range of conditions and diagnoses associated with pediatric trauma patients varies widely. Despite the concern for ACD in the aforementioned studies, etomidate use seems to be common among pediatric trauma programs in the United States. While clinicians can diagnose sepsis and shock upon presentation, it is rather difficult to know which, if any, trauma patients will progress to meeting sepsis and/or shock criteria after initial presentation. Interestingly, approximately 65% of respondents do not believe ACD is clinically relevant, specifically in pediatric trauma patients. However, none of the respondents check cortisol levels in any of their pediatric trauma patients to assess whether ACD is present or significant.

Typically, there are several questions that clinicians feel need to be answered regarding etomidate. The first is whether etomidate inhibits cortisol production. Pharmacologically, inhibition of 11-beta-hydroxylase occurs after etomidate administration. Secondly, does this inhibition lead to detectable decreases in cortisol in pediatric patients? There appears to be detectable decreases after etomidate administration but in pediatrics. However, observational study methodology makes this difficult to discern [41]. Lastly, is the inhibition of cortisol clinically relevant? This question is more difficult to answer based on the data currently available in pediatrics. With the uncertainty in the clinical significance of pediatric ACD and its potential for harm through cortisol suppression, why is it so broadly accepted when other, safer alternatives that can be used in this setting?

## 5. Limitations

Limitations of our survey and the generalizability of its findings include a lower response rate, which introduces the potential for non-response bias. A small sample of respondents could limit the interpretation of any significant relationship for the use of etomidate for RSI in certain clinical scenarios. In order to develop a survey that practitioners would be able to complete in a timely manner, the survey was only able to query basic information regarding the use of etomidate for RSI for pediatric trauma patients. Interest in the use and potential consequences of etomidate in the pediatric trauma population could be heightened through the creation of a working group in a professional society and/or a practice based research network (PBRN). More detailed information, perhaps a medication use evaluation (MUE), could be conducted with a larger, more in-depth survey. Respondent bias could also have affected the response rate in this survey because clinicians who do not use etomidate may not be as likely to complete the survey as are clinicians who do use etomidate. There may also be differences between self-reported practice and actual practice. Further, as an exploratory pilot study, the purpose of the survey was to evaluate the feasibility of conducting future research on etomidate use in pediatric trauma patients. Assessing the practical aspects of carrying out a larger scale survey and/or prospective clinical research study is paramount.

## 6. Conclusions

In this self-selected sample, the use of etomidate in pediatric trauma patients undergoing RSI is common among urban, academic, teaching, level 1 pediatric trauma centers. A retrospective MUE or case series coupled with a prospective evaluation of etomidate use for RSI in pediatric trauma patients to evaluate its potential ACD, hemodynamics, and outcomes is warranted.

## Footnotes

1 Location: St. Christopher’s Hospital for Children (SCHC) is a 189-bed freestanding children’s tertiary care teaching hospital in Philadelphia, PA that is a Level 1 trauma center with 33 critical care beds that provide care for children with a variety of critical illness including burns, trauma, congenital heart disease and children on extra corporeal life support (ECLS).

## Appendix 1. Survey Questions

Use of Etomidate for Rapid Sequence Intubation (RSI) in Pediatric Trauma Patients: A National Survey.

(1) Select the geographic region for which you program is located:
  - Northeast
  - Southeast
  - Midwest
  - Southwest
  - West coast
(2) What is the certification of your trauma center?
  - Level 1 Pediatric
  - Level 2 Pediatric
  - Level 3 Pediatric
  - Level 1 Adult Trauma Center with Pediatric certification
  - Level 2 Adult Trauma Center with Pediatric certification
  - Level 3 Adult Trauma Center with Pediatric certification
(3) Which of the following best describes your trauma center (select all that apply);
  - Academic
  - Non-academic
  - Teaching
  - Non-teaching
  - Urban
  - Rural
(4) What is your role in the trauma program?
  - Trauma Program Director
  - Trauma Program Coordinator
  - Anesthesiologist
  - Emergency Department Director
  - Critical Care
(5) For which of the following do you provide care?
  - Adults patients only
  - Adult and pediatric patients
  - Pediatric patients only
(6) On average, how many pediatric trauma patients do you provide care for annually?
  - <50
  - 50–100
  - 100–200
  - >200
(7) On average, how many pediatric trauma patients that you provide care for are intubated within your facility annually?
  - <10
  - 10–24
  - 25–49
  - >50
(8) Where are your pediatric trauma patients primarily cared for?
  - Separate Trauma bay
  - Trauma Area within the general Emergency Department
  - General Emergency Department bed
(9) Who is responsible for the airway management in your pediatric trauma patients?
  - Surgery
  - Critical Care
  - Emergency Department
  - Anesthesia
(10) Do you have a protocol for RSI in pediatric trauma patients?
  - Yes
  - No
(11) When was the last revision to your RSI protocol?
  - <6 months ago
  - 6 months–1 year ago
  - 2–4 years ago
  - ≥5 years ago
  - Don’t know
(12) Who sets the RSI protocol for your pediatric trauma patients?
  - Trauma
  - Surgery
  - Critical Care
  - Emergency Department
  - Anesthesia
(13) Do you have a separate policy regarding RSI in pediatric trauma patients?
  - Yes
  - No
(14) What medications do you commonly utilize for RSI in your pediatric trauma patients?
  - Answer entry
(15) Do you use etomidate for RSI in your pediatric trauma patients?
  - Yes
  - No
(16) Is etomidate used in all pediatric trauma patients, regardless of etiology or nature of the trauma?
  - Yes
  - No
  - If no, who is excluded; answer entry
(17) Are you aware of the side effects associated with etomidate?
  - Yes
  - No
(18) Do you believe that etomidate is associated with adrenal suppression?
  - Yes
  - No
(19) If you use etomidate for RSI, in your opinion, what is the primary advantage of using etomidate?
  - Rapid onset
  - Permits neurologic evaluation sooner than most sedatives
  - Minimal effect on hemodynamics
  - Drug of choice for traumatic brain injury
  - Good analgesic properties
  - Other: please fill in
(20) If you do not use etomidate for RSI, in your opinion, what is the primary disadvantage of using etomidate?
  - Nausea/vomiting
  - Laryngospam
  - Myoclonus
  - Adrenal suppression
  - Other: please fill in
(21) Do you believe the use of etomidate can exacerbate the adrenal suppression seen in pediatric trauma patients?
  - Yes
  - No
(22) In your opinion, do you believe that the adrenal suppression associated with etomidate is a clinically relevant concern in your pediatric trauma patients?
  - Yes
  - No
(23) If you use etomidate in pediatric trauma patients, under what circumstances would you consider altering your practice?
  - Answer entry
(24) If you use etomidate in pediatric trauma patients, do you routinely obtain cortisol levels?
  - Yes
  - No
  - If yes why: answer entry
(25) If you obtain cortisol levels, what is your cortisol threshold level for using replacement hydrocortisone therapy?
  - <10 mg/dL
  - <15 mg/dL
  - <18 mg/dL
  - <25 mg/dL
  - Other; please fill in
